# Enabling Radiation Hardness in Solid-State NAND Storage Utilizing a Laminated Ferroelectric Stack

**DOI:** 10.1021/acs.nanolett.5c05947

**Published:** 2026-03-05

**Authors:** Lance Fernandes, Stuart Wodzro, Prasanna Venkatesan, Priyankka Ravikumar, Ming-Yen Lee, Minji Shon, Dyutimoy Chakraborty, Taeyoung Song, Sanghyun Kang, Salma Soliman, Mengkun Tian, Jason Yeager, Jackson Adler, Jiayi Chen, Zekai Wang, Douglas Wolfe, Shimeng Yu, Andrea Padovani, Suman Datta, Biswajit Ray, Asif Khan

**Affiliations:** † School of Electrical and Computer Engineering, 1372 Georgia Institute of Technology, Atlanta, Georgia 30332, United States; ‡ Institute of Matter and Systems, Georgia Institute of Technology, Atlanta, Georgia 30332, United States; § Department of Material Science and Engineering, 8082 Pennsylvania State University, University Park, Pennsylvania 16802, United States; ∥ Department of Engineering Sciences and Methods, 9306 University of Modena and Reggio Emilia, 41121 Reggio Emilia, Italy; ⊥ Department of Electrical and Computer Engineering, 3447 Colorado State University, Fort Collins, Colorado 80523, United States

**Keywords:** ferroelectric NAND, γ radiation, reliability, large memory window

## Abstract

NAND flash forms the core of modern solid-state storage, which is critical for data-intensive AI applications, yet charge-trap NAND suffers rapid threshold-voltage (*V*_th_) degradation under ionizing radiation, causing reliability challenges for space and defense applications. Here we show that ferroelectric field-effect transistors (FeFETs) with laminated gate stacks offer a promising route to achieving radiation resilience in vertical NAND technology. We demonstrate that large-memory-window, vertical NAND-compatible laminated poly-silicon-channel FeFETs with an 8 nm Hf_0.5_Zr_0.5_O_2_/3 nm Al_2_O_3_/8 nm Hf_0.5_Zr_0.5_O_2_ stack retain a full memory window and robust switching up to 10 Mrad­(air) of the total ionizing dose (TID). Programmed and erased states show negligible TID-induced drift after 1 Mrad­(air), while only the erased state degrades by ∼2 V at 10 Mrad­(air). Technology computer-aided design (TCAD) modeling attributes these asymmetric shifts to state-dependent traps. Compared to charge-trap NAND, laminated FeFETs exhibit ∼30-fold lower *V*_th_ degradation per unit dose, positioning them as superior radiation-resilient storage candidates.

## Graphical abstract

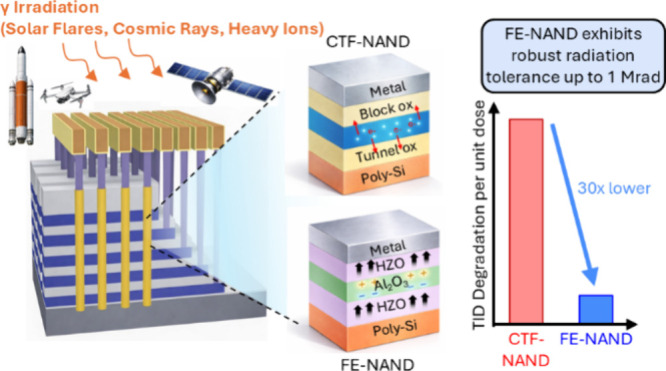

NAND flash forms the foundation of solid-state storage, driving applications from consumer electronics to cloud infrastructure and emerging data-intensive workloads that demand massive, reliable, and ultra-high-density (approximately gigabits per square millimeter) memory capacity. Reliable nonvolatile memory in radiation-rich environments is vital for space, aerospace, and defense electronics, where even minor data corruption can jeopardize mission-critical operations. However, state-of-the-art 3D NAND flash, based on charge-trap (CTF) or floating gate (FG) storage, is highly susceptible to total ionizing dose (TID) effects. Irradiation generates electron–hole pairs within the oxide stack and accelerates defect formation, leading to charge loss.^1–3^ In programmed cells, stored electrons are depleted through photoemission, recombination between stored electrons and TID-induced holes, and hole trapping in the gate dielectrics, reducing the threshold voltage (*V*_th_) and ultimately causing bit-flip errors.^1–4^ Such failures arise at doses as small as 50 krad, far below typical space conditions, underscoring the inadequacy of conventional NAND for extreme environments.^2^ While magnetic storage offers radiation tolerance, it cannot meet the stringent size, weight, and power (SWaP) constraints. Alternative nonvolatile memories such as ferroelectric random access memory (FeRAM), magnetic random access memory (MRAM), and resistive random access memory (RRAM) exhibit excellent ionizing radiation tolerance;^5–8^ however, their limited bit density, typically in the range of megabits per square millimeter, prevents them from serving as mass storage-class memories, which require ultrahigh areal bit densities (gigabits or terabits per square millimeters) comparable to NAND flash for mass data storage. Therefore, the development of semiconductor-based, radiation-resilient nonvolatile mass storage-class memory that is inherently immune to irradiation is essential for future systems.

Ferroelectric field-effect transistors (FeFETs) based on hafnia oxides have emerged as promising alternatives to charge-based flash.^9,10^ By storing information in remanent polarization rather than trapped charge, FeFETs inherently mitigate many of the failure modes associated with radiation exposure. In addition, FeFETs enable low-voltage operation, fast switching, and vertical scalability, making them attractive for future 3D NAND architectures.^9^ However, HZO-only FeFETs exhibit a limited memory window (MW) due to thickness constraints, rendering them unsuitable for vertical NAND storage applications.^10^ Recent studies have demonstrated that introducing a dielectric layer either between stacked HZO layers or between the ferroelectric and metal gate forms laminated ferroelectric/dielectric (FE/DE) stacks that significantly expand the MW at comparable thicknesses by leveraging interfacial charge at the FE–DE boundary.^10–15^ The analytical memory window (MW) equation responsible for the MW enhancement has been derived in previous works^10,15,38^ and is shown in eq 1

$$\mathrm{MW}=\frac{\Delta \left(P_{r}-Q_{it}\right)}{C_{FE}}+\frac{\Delta \left(Q_{\mathrm{it\prime}}-Q_{it}\right)}{C_{\mathrm{DE}}}$$ 1

where *Q*_it_ denotes the trap charges at the channel-side interlayer and *Q*_it′_ represents the interfacial trap charges at the ferroelectric–dielectric (FE–DE) interface. Moreover, as shown in Figure S3.2, the laminated gate stack enhances the ferroelectric properties, increasing the remanent polarization (2*P*_r_) and coercive voltage (2*V*_c_) by approximately 1.5- and 2-fold, respectively, compared to the HZO-only stack.

This architecture supports multilevel operation compatible with vertical NAND technology while retaining strong retention, positioning laminated FeFETs as a promising platform for high-density, reliable vertical NAND storage.^14–21^

Despite these advantages, the effect of the TID on laminated FeFETs remains largely unexplored. Previous radiation studies have focused mainly on Hf_0.5_Zr_0.5_O_2_ (HZO)-only gate stacks, examining threshold-voltage shifts and memory window degradation under irradiation.^22–25^ However, HZO-only FeFETs lack the large memory window needed for vertical NAND technology. Large-memory-window (MW) laminated FE/DE stacks introduce additional interfaces and complex trap dynamics that lead to MW enhancement compared to HZO-only stacks. Gaining a clear understanding of these mechanisms and their impact on the irradiation performance is critical to evaluating the suitability of laminated FeFETs for operation in extreme environments.

In this work, we systematically investigate laminated poly-Si FeFETs with an 8 nm HZO/3 nm Al_2_O_3_/8 nm HZO gate stack exposed to ^60^Co γ-irradiation up to 10 Mrad­(air) with a dose rate of 112 krad/h­(air). The irradiation experiment was conducted by using air (atmospheric conditions) as the reference medium. For the first time, we demonstrate that programmed and erased states in high-MW laminated poly-Si FeFETs remain stable up to 1 Mrad­(air) TID, exhibiting a negligible TID-induced *V*_th_ shift and markedly superior radiation immunity compared to CTF flash, a key requirement for radiation-hardened memory applications. This results in achieving ∼30-fold lower normalized TID-induced *V*_th_ degradation compared to state-of-the-art CTF NAND.^1–3^ At a TID exposure of 10 Mrad­(air), the erased state is degraded, while the programmed state remains largely unaffected. Moreover, we show that the maximum achievable MW shows a negligible change even at a TID dose of 10 Mrad­(air). Furthermore, calibrated TCAD modeling provides insight into the physical origins of state-dependent *V*_th_ shifts, revealing the critical role of trap formation near the channel interlayer in the erased state. These findings establish laminated FeFETs as a strong candidate for radiation-hardened 3D NAND technologies, combining vertical NAND-compatible MW and robust retention along with exceptional radiation resilience comparable to previously reported single-layer HZO-only FeFETs.^22–25^ This work investigates the effects of radiation on ferroelectric NAND stack-based devices, focusing exclusively on the memory cell structures and excluding the supporting CMOS circuitry, which is typically more susceptible to radiation-induced degradation.

The fabrication process flow for the poly-Si channel FeFET with a laminated stack is presented in section S1 of the Supporting Information. The TEM image of the laminated stack with EDS elemental mapping is shown in section S2.

The devices were initialized in programmed and erased states. A control sample was left unirradiated, while two others were subjected to a TID of 1 and 10 Mrad­(air). The measurement scheme is illustrated in Figure 1a. The device *V*_th_ was first extracted immediately (1 s read delay) after programming and/or erasing to establish the initial state. The control samples with stored states were measured after room-temperature storage for 30 days to capture only depolarization effects. In contrast, the irradiated samples were exposed to 1 and 10 Mrad­(air) after the initial *V*_th_ extraction, and then the initially stored states were measured again after 30 days, incorporating both depolarization and TID effects. As shown in Figure 1a, the first 2 weeks correspond to standard retention under ambient conditions while the subsequent 2 weeks represent post-irradiation retention behavior at 1 and 10 Mrad­(air), totaling approximately 30 days. We note that irradiation and electrical characterization were performed ex situ, which may have led to a partial room-temperature recovery of radiation-induced damage. However, the same measurement protocol, with an identical post-irradiation time line, was used for all samples, ensuring comparable room-temperature recovery. Therefore, the observed differences between 1 and 10 Mrad can be attributed to the absorbed dose rather than to recovery effects.

**Figure 1:**
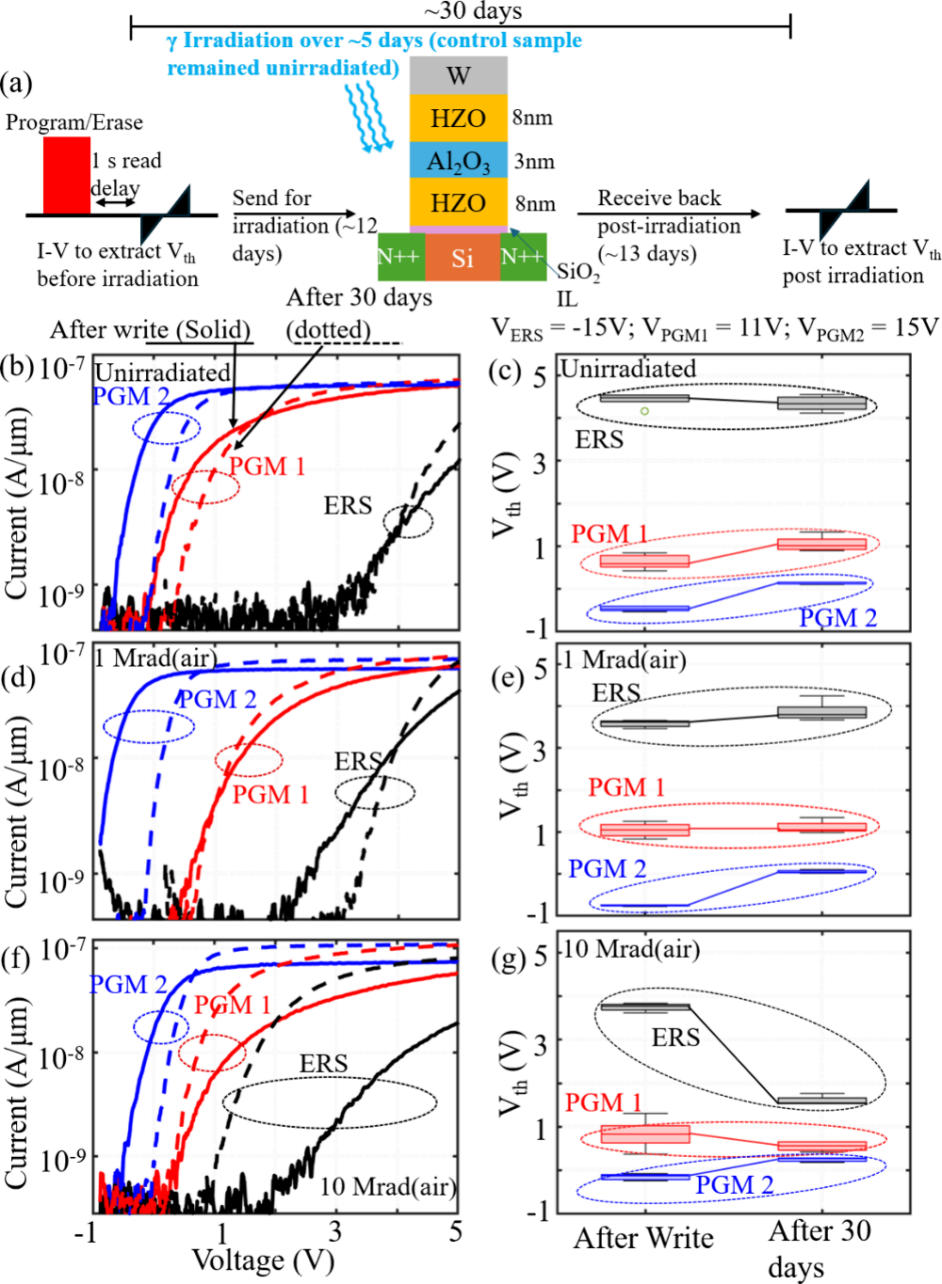
Survivability of *V*_th_ states under irradiation. (a) *V*_th_ measurement scheme used to evaluate total ionizing dose (TID)-induced *V*_th_ loss for control (unirradiated) and irradiated samples. The control device was stored under ambient conditions, while the other samples were programmed and subsequently exposed to irradiation. (b and c) *I–V* and *V*_th_ distribution, respectively, measured for the control (unirradiated) sample immediately after the write operation (1 s delay) and after ambient storage for 30 days (standard retention). The solid curves represent the *I–V* plot after writing, whereas the dotted curves correspond to the *I–V* plot after 30 days. (d–g) *I–V* and *V*_th_ distribution measured immediately after the write operation (1 s delay) and after a total of ∼30 days, which include ∼2 weeks of standard retention followed by ∼2 weeks after exposure to 1 and 10 Mrad­(air) TID, respectively. The solid curves represent the *I–V* plot immediately after writing, while the dotted curves correspond to measurements taken 30 days after writing and post-irradiation. The write voltages for the erased (ERS), programmed state 2 (PGM 2), and programmed state 1 (PGM 1) operations are −15, 15, and 11 V, respectively, each with a 10 μs pulse width. Each *V*_th_ distribution shown here is at least five data points.

As shown in panels b and c of Figure 1, for the unirradiated sample, the erased-state *V*_th_ exhibits minimal degradation, while the programmed states (state 1 and state 2) show only a modest increase (∼0.5 V for the programmed state), attributed to the depolarization field effects reported previously.^26^ Panels d and e of Figure 1 further reveal that after 1 Mrad­(air), both programmed- and erased-state *V*_th_ values shift by amounts comparable to the control (unirradiated) case, indicating negligible TID-induced *V*_th_ loss. The erased-state *V*_th_ values in panels d and e of Figure 1 show a slight increase compared to those of the initial state, which may be attributed to the read-after-write delay commonly observed in conventional FeFETs.^27^ However, no TID-induced degradation is observed up to 1 Mrad­(air). At 10 Mrad­(air), however, the erased state degrades significantly due to TID (∼2 V, ∼8-fold larger than the control or 1 Mrad­(air)), as shown in panels f and g of Figure 1. The fully programmed-state (PGM2) threshold voltage (*V*_th_) shifts positively after 30 days for both unirradiated and irradiated samples, regardless of the TID level, as shown in panels b–g of Figure 1. Importantly, the magnitude of this positive shift in the programmed state is comparable for unirradiated and irradiated samples and remains in the range of 0.5–0.8 V. This indicates that TID does not introduce an additional shift in the programmed state beyond that observed in the unirradiated control sample. Accordingly, the fully programmed-state *V*_th_ shift can be attributed to depolarization field-induced *V*_th_ loss, which is commonly observed in FeFETs^26^ with no additional TID-induced degradation. These results further suggest that TID-induced degradation manifests predominantly in the erased state, and only at extreme doses of 10 Mrad­(air), while the fully programmed state remains free of TID-induced degradation even at large doses, exhibiting only depolarization-related effects.^26^ Alternative gate stack engineering schemes need to be explored to mitigate the depolarization effects in ferroelectric materials^28^ to further reduce the depolarization-induced *V*_th_ loss. These results are in alignment with previous reports on the HZO-only stack.^22,24^ The results suggest that Fe-NAND has a 30-fold lower TID-induced *V*_th_ shift (∼0.25 mV/krad) compared to state-of-the-art CTF NAND, which shows *V*_th_ degradation of ∼0.4 V at TIDs as low as 50 krad (8.5 mV/krad).^1–3^ While the fully programmed states (PGM2) remain free of TID-induced degradation and exhibit only depolarization field-induced loss, the partially programmed states (PGM1) show a pronounced negative threshold-voltage shift at 10 Mrad­(air) that is absent in both unirradiated and 1 Mrad samples, as shown in Figure 1f. This indicates that fully programmed states are resilient to TID, whereas partially programmed states become susceptible at large doses; the underlying mechanism is discussed below.

Moreover, the slight differences between the absolute post-write threshold-voltage distribution observed in panels c, e, and f of Figure 1 among the PGM2 states for unirradiated and irradiated samples arise from sample die-to-die variability across fabrication batches. However, TID-induced effects on the stored states are assessed based on the relative threshold-voltage shift with respect to the initial post-write state of the same device, rather than the absolute post-write or post-irradiation *V*_th_ values. As a result, die-to-die variability in the absolute *V*_th_ distribution does not affect the validity of the analysis, since the relative *V*_th_ shift is the physically relevant metric for evaluating TID-induced changes in the stored state. Additionally, the subthreshold swing (SS) of both unirradiated and irradiated devices is observed to degrade when measured immediately after program/erase operation (postwrite). However, the SS improves significantly after ambient aging for 30 days, approaching similar values for both unirradiated and irradiated samples. Immediately after high-field program/erase pulses, transient injected carriers and metastable trapped charge in the oxide and channel interlayer increase the effective trap capacitance, leading to a degraded SS. During subsequent ambient storage, partial detrapping, recombination, and redistribution of these charges result in room-temperature relaxation and recovery of the SS. Importantly, this recovery behavior is observed for both unirradiated and irradiated devices, as shown in panels b, d, and f of Figure 1 for both programmed and erased states, indicating that the SS degradation after write and its subsequent recovery are predominantly a transient effect associated with write-induced charge trapping rather than a radiation-induced mechanism. The SS relaxation in irradiated devices postirradiation reflects a time-dependent trap relaxation rather than the absence of radiation-induced defect generation, as more permanent traps may persist.

To estimate the performance for NAND application after TID, systematic measurements were performed under identical conditions on unirradiated and irradiated samples. Pulsed *I–V* measurements (Figure 2a) were performed; a pristine device was programmed and/or erased, and the device’s *V*_th_ state was measured at 20 nA × W/L after a read delay of 1 s. As shown in Figure 2b, the device *I–V* plot after program/erase shifts to the right as the TID dose increases compared to the unirradiated sample. Figure 2c shows the programmed (LVT) and erased (HVT) *V*_th_ distribution extracted from the pulsed *I–V* plot. The maximum achievable memory window remains nearly unchanged across all doses, as shown in Figure 2c. This indicates that the device’s fundamental ability to retain a full MW is largely unaffected by irradiation. While interface traps at the FE–DE interface in laminated HZO stacks can strongly influence ferroelectric properties and a memory window (eq 1), the maximum achievable MW remains nearly unchanged across control and irradiated devices. This stability indicates minimal variation in the effective interfacial trap density within the investigated TID range. Panels d and g of Figure 2 illustrate the pulse schemes used for incremental step pulse programming (ISPP) and incremental step pulse erase (ISPE), respectively, which are standard techniques in NAND architectures. In ISPP/ISPE, the program or erase voltage is applied in small incremental steps rather than a single large pulse, enabling precise tuning of the threshold voltage (*V*_th_) for accurate multilevel (MLC/TLC/QLC) operation while minimizing overprogramming, program/erase disturb, and cell-to-cell variability. The resulting ISPP and ISPE characteristics for control and irradiated devices are shown in panels e and h, respectively, of Figure 2, with the corresponding average slopes summarized in panels f and i, respectively, of Figure 2. A higher ISPP/ISPE slope indicates a more efficient *V*_th_ increase per program/erase step, leading to faster programming/erasing and reduced cumulative stress on the gate stack. As TID increases, the ISPP and ISPE slopes decrease, demonstrating a reduction in program and erase efficiency under irradiation. In practical NAND architectures, dose-adaptive erase-verify algorithms and read-reference calibration based on the absorbed TID can be used to compensate for the relative *V*_th_ shifts that develop after program/erase operations at larger radiation doses.

**Figure 2:**
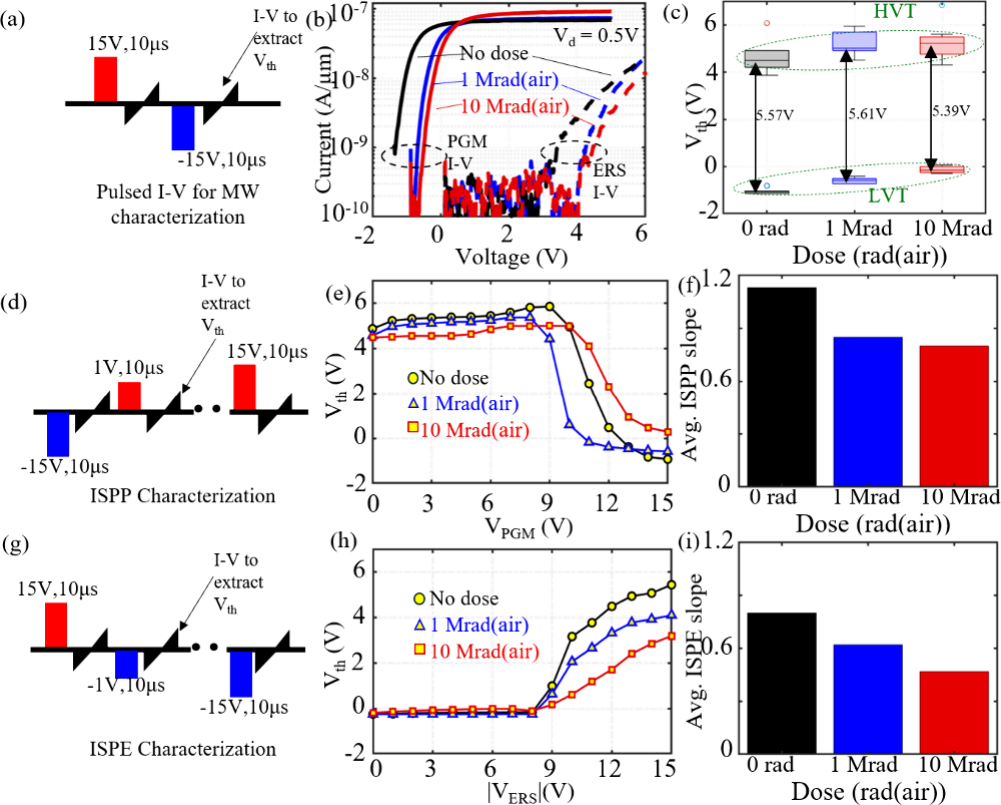
Performance characterization for unirradiated and irradiated samples. (a) Pulsed *I–V* measurement scheme used for memory-window (MW) extraction. (b) Pulsed *I–V* characteristics of programmed and erased states for unirradiated, 1 Mrad­(air) TID-exposed, and 10 Mrad­(air) TID-exposed devices, used to extract *V*_th_ immediately after writing (1 s read delay). (c) *V*_th_ distributions for programmed and erased states from a pulsed *I–V* plot under the same write conditions. (d) Incremental step pulse programming (ISPP) scheme. (e) Evolution of *V*_th_ as a function of programming pulse amplitude (*V*_PGM_) for control and irradiated samples. (f) Average ISPP slope for unirradiated, 1 Mrad­(air) TID, and 10 Mrad­(air) TID conditions. (g) Incremental step pulse erase (ISPE) scheme. (h) Evolution of *V*_th_ as a function of the erase pulse amplitude (|*V*_ERS_|) for control and irradiated samples. (i) Average ISPE slope for unirradiated, 1 Mrad­(air) TID, and 10 Mrad­(air) TID conditions.

Figure 3b shows the results of standard retention measurements (measurement scheme shown in Figure 3a) performed on unirradiated and irradiated samples up to 10^4^ s, showing robust retention. The MW loss at 10^4^ s increases with TID as shown in Figure 3c, highlighting the effect of TID on retention of device *V*_th_ states. This retention loss primarily arises from detrapping of FE/Al_2_O_3_ trap charges due to TID-induced defects. These trap charges are responsible for MW enhancement, and detrapping of these charges leads to an increase in MW loss with TID.^19,20,29^ Pass disturb measurements are used to evaluate how the stored state of unaccessed cells in a NAND string is affected when a pass voltage is applied to turn them on during the access of a selected cell. To investigate this effect, pass disturb experiments were performed on both unirradiated and irradiated devices to assess the impact of repeated pass disturb cycles on the programmed and erased states, as shown in Figure 3d. Ideally, pass disturb cycles should induce minimal shifts in the device threshold voltage (*V*_th_). In this work, the pass disturb voltage was set to HVT + 0.5 V, corresponding to approximately 6 V, which is sufficiently high to ensure that unaccessed cells are fully turned on during NAND string operation. To perform the measurement, the device was first initialized to either the programmed (LVT) or erased (HVT) state using a write pulse, followed by the application of pass disturbance cycles, as illustrated in Figure 3d. After a specified number of disturbed cycles, an *I–V* read was performed to extract the resulting *V*_th_ shift. As the pass disturb cycle increases, electrons are trapped in channel IL, which consequently increases the device *V*_th_ on the LVT side, as shown in Figure 3e. The shift in LVT after 10^6^ disturb cycles is plotted in Figure 3f, highlighting a slight increase in the LVT shift for samples exposed to 10 Mrad­(air) TID. The shift in LVT for control and the 1 Mrad­(air)-exposed sample is comparable, suggesting no additional TID-induced *V*_th_ shift on the LVT side due to pass disturb cycles.

**Figure 3:**
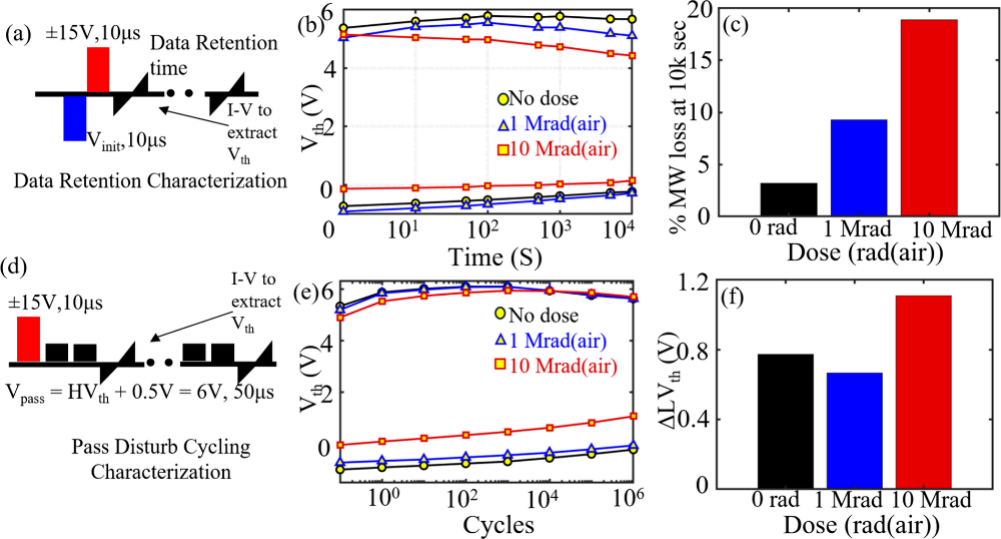
Retention and disturb characterization for unirradiated and irradiated samples. (a) Retention characterization measurement scheme. (b) Standard retention measurements on control and irradiated devices. (c) Memory window (MW) loss at room temperature after 10^4^ s as a function of TID dose. (d) Measurement scheme incorporating pass disturb cycling after write operation to estimate the shift in *V*_th_ after disturb cycles. (e) Evolution of programmed and erased *V*_th_ under pass disturb conditions for control and irradiated samples. (f) Shift in the programmed (low) *V*_th_ state after 10^6^ pass disturb cycles vs TID dose.

To further elucidate the mechanisms of TID effects in laminated stacks, Sentaurus TCAD simulations were performed and calibrated to the experimental data. The memory window enhancement in the laminated stack is modeled using a Preisach ferroelectric polarization framework to capture remnant polarization switching, combined with fixed interface traps at the HZO/Al_2_O_3_ interfaces, which was previously modeled by Shon et al.^30^ Radiation effects were modeled using Sentaurus TCAD’s built-in γ irradiation module, which computes the electron–hole pair generation rate within each material layer based on the experimentally applied dose rate and exposure duration for both programmed and erased steady states. The resulting local energy deposition, representing the energy transferred from incident radiation to the material per unit mass, was used to obtain the cumulative electron–hole generation, which was then mapped to spatial trap distributions induced by the TID across the gate stack through a calibrated efficiency factor. This approach effectively reproduces the experimentally observed threshold-voltage shifts induced by the total ionizing dose (TID) exposure. Panels a and b of Figure 4 show band diagrams for unirradiated and irradiated cases in the HVT (erased) and LVT (programmed) hold states, respectively. Ionizing radiation generates electron–hole pairs within the gate stack, which redistribute under the built-in ferroelectric polarization field (depolarization). In the HVT state (Figure 4a), the depolarization field points toward the channel with holes drifting and accumulating near the channel interlayer (IL), which degrades the HVT state.^24,31,32^ In the LVT state (Figure 4b), the depolarization field points away from the channel interlayer (IL), causing electrons to drift toward the IL under the built-in (depolarization) electric field. These electrons can readily detrap and redistribute due to their high mobility, resulting in a minimal TID-induced *V*_th_ shift for fully programmed states even at high TID.^32^ This carrier redistribution behavior is characteristic of conventional HZO-only (FE-only) stacks, as reported previously,^31,32^ and serves as the reference for comparison with the laminated stack studied here. Moreover, the partially programmed intermediate state (PGM1) (Figure 1b,d,g) exhibits a reduced and spatially nonuniform polarization compared to fully programmed and erased states. Although the net polarization magnitude is smaller than that of the erased state, the resulting depolarization field can still point toward the channel, albeit with a reduced strength. Consequently, radiation-generated holes can migrate toward and accumulate near the channel interlayer, but with reduced accumulation compared to the erased state leading to a measurable negative threshold-voltage shift at extreme TID observed in Figure 1f for the partially programmed (PGM1) state. This state-dependent carrier transport and trapping behavior explains the divergence observed between PGM1 and PGM2 in Figure 1 at large radiation doses and confirms that TID-induced degradation predominantly affects intermediate and erased states, while the fully programmed state is mainly affected by depolarization field loss.

**Figure 4:**
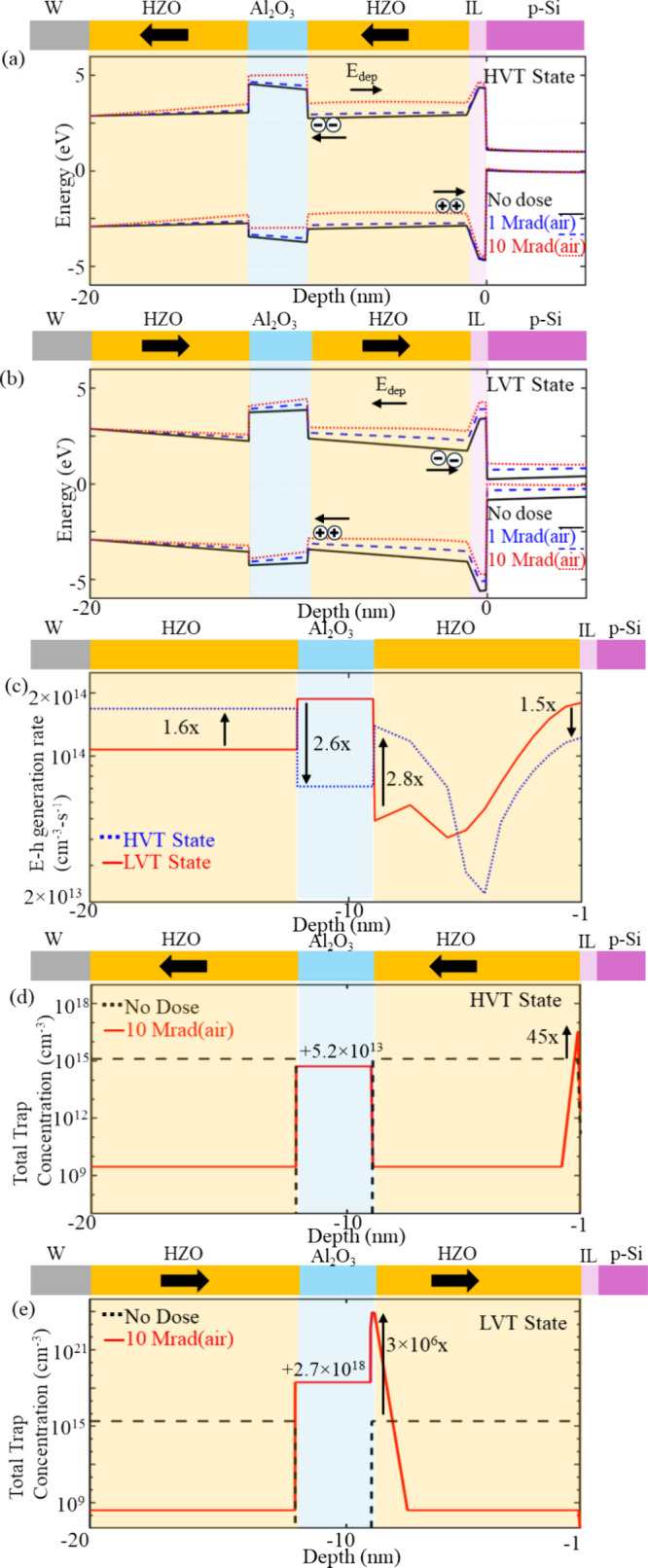
Modeling the irradiation mechanism with TCAD. (a and b) Band diagrams of devices in erased (HVT) and fully programmed (LVT) hold states, respectively, extracted from TCAD simulations calibrated to experimental trends. (c) Calculated electron–hole pair generation rate at a TID dose rate of 112 krad/h for the LVT and HVT states. (d and e) Total TID-induced trap concentration (acceptor and donor) before and after 10 Mrad­(air) irradiation for the HVT and LVT states, respectively.

Additionally, the introduction of an Al_2_O_3_ layer modifies the built-in electric (depolarization) field within the oxide differently for the LVT and HVT hold states with an increase in TID dose compared to the HZO-only stack. As shown in panels a and b of Figure 4, the TID-induced electron–hole pairs redistribute and modify the internal electric fields differently. As one can clearly see from the changes in band slope (reflecting band bending due to the internal electric field) with an increase in TID in panels a and b of Figure 4, the HVT state exhibits a primary reduction of the built-in electric field across the Al_2_O_3_ dielectric (pronounced changes in the band slope in the Al_2_O_3_ region), whereas in the LVT state, pronounced changes occur in the built-in depolarization field across the bottom ferroelectric layer and the channel-side interlayer. Moreover, the changes observed in the top HZO layer are similar for both HVT and LVT states, which is evident from the minimal change in band slope with an increase in TID.

While the ferroelectric polarization itself is relatively stable under irradiation, the HZO layer, like other oxide dielectrics, remains susceptible to radiation-induced electron–hole pair generation and subsequent charge trapping under γ irradiation. Figure 4c presents the spatially nonuniform, state-dependent e–h generation rate under irradiation at 112 krad/h. The electron–hole pair generation rate calculated using Sentaurus TCAD’s γ irradiation model is based on local radiation energy deposition, material-specific generation efficiency, and the applied dose rate and exposure. In the HVT state, electron–hole pair generation is higher within the top ferroelectric layer and near the Al_2_O_3_–ferroelectric interface, leading to an increase in radiation-induced charge pairs in these regions. This results in radiation-induced charge accumulation that predominantly screens and relaxes the electric field across the Al_2_O_3_ dielectric, which is evident from band bending with an increase in TID in Figure 4a for the HVT state. In the LVT state, electron–hole pair generation is concentrated within the Al_2_O_3_ layer and near the channel-side interlayer, causing charge redistribution in these regions that primarily relax the electric field across the bottom ferroelectric layer. In the model, the local TID-induced trap concentration is proportional to the spatial radiation dose map (the spatial variation in energy deposition, which affects the electron–hole generation rate). A region-dependent efficiency factor was introduced to account for variations in trap generation across different layers of the gate stack. This factor maps the cumulative locally generated electron–hole pairs for a given dose and material to the fraction of charges that become trapped, thereby producing the observed device *V*_th_ shift. By tuning this efficiency factor, the model reproduced the experimentally observed threshold-voltage shifts under TID exposure. As shown in Figure 4d, the total TID-induced trap concentration (TTC) in HVT increases by ∼45-fold (compared to the reference trap concentration in unirradiated case) near the IL, with additional TID-induced traps generated in Al_2_O_3_ (∼5.2 × 10^13^) compared to the unirradiated state. The holes generated during irradiation drift toward the IL and become trapped in the IL-proximal states, explaining the experimentally observed degradation of the HVT state at large doses. In contrast, as shown in Figure 4e, for the LVT state, new traps are generated predominantly within Al_2_O_3_ (∼2.7 × 10^18^), and TTC near the bottom FE/Al_2_O_3_ interface increases by ∼3 × 10^6^ at 10 Mrad­(air) while negligible trap formation occurs near the IL. These results highlight that TID-induced traps in Al_2_O_3_ or the FE–Al_2_O_3_ interface have little impact on *V*_th_ stability, whereas trap generation near the IL that occurs only in the erased state is the dominant contributor to erased-state degradation. In the programmed state, trap formation near the channel interlayer (IL) is negligible and therefore does not contribute to any TID-induced *V*_th_ degradation; the observed *V*_th_ shift in programmed state arises solely from the intrinsic depolarization field.

Table 1 shows the landscape of different nonvolatile memory technologies and its corresponding radiation tolerance with areal bit density. The radiation tolerance reported here is the intrinsic tolerance of the memory device without considering the supporting CMOS periphery, which fails at relatively smaller TID doses. Ferroelectric NAND combines a mass storage capability with a high radiation tolerance.

**Table 1:** Radiation Tolerance Landscape of Nonvolatile Memories^a^

memory technology	typical bit density	storage type	reported radiation tolerance
ferroelectric RAM (FeRAM)	Mb/mm^2^ ^33–35^	embedded nonvolatile storage/storage class	∼10 Mrad^7^
magnetic RAM (MRAM)			∼10 Mrad^5,6^
resistive RAM (ReRAM)			>5 Mrad^8^
charge-trap-flash (CTF) NAND	Gb/mm^2^ ^36,37^	mass storage	∼50 krad (0.4–0.5 V V_th_ loss)^1–4^
ferroelectric NAND (Fe-NAND)	Gb/mm^2^ (projected for 3D arrays, compatible with CTF NAND technology)		1 Mrad

a Mass storage-class technologies (CTF NAND, and laminated Fe-NAND) are compared with embedded-class NVMs. Radiation tolerance values reflect only the intrinsic memory device and not the supporting CMOS periphery, which typically fails at a much lower TID. Laminated Fe-NAND uniquely combines mass storage-class density with 1 Mrad­(air) TID tolerance.

In conclusion, in this work, we have demonstrated that large-memory-window laminated ferroelectric-gated poly-silicon FeFETs with an HZO/Al_2_O_3_/HZO stack exhibit unprecedented resilience against total ionizing dose (TID) radiation of up to 1 Mrad­(air). Experimental results revealed that the programmed states remain largely unaffected by TID even under large-dose irradiation, while the erased state shows TID-induced degradation only at an extreme dose of 10 Mrad­(air), attributable to trap formation near the channel interlayer. The observed erase *V*_th_ loss at 10 Mrad­(air) suggests that data refresh mechanisms may be needed for reliable operation above 1 Mrad­(air). The maximum achievable memory window and ferroelectric switching characteristics remain unchanged even at 10 Mrad­(air) TID. While the FeFETs retain a strong memory window, both the programmed and erased threshold voltages shift with TID, accompanied by a degradation in the ISPP/ISPE slopes. These shifts can be corrected by adjusting read reference voltages and erase verification schemes based on absorbed TID. TCAD modeling provided physical insight into the observed state-dependent *V*_th_ shifts, identifying the role of trap formation at the interface and redistribution of electron–hole pairs within the oxide stack. Compared with state-of-the-art charge-trap NAND flash, laminated FeFETs exhibit a nearly 30-fold improvement in TID tolerance, positioning them as strong candidates for nonvolatile storage in space, aerospace, and defense applications. However, system-level challenges remain. A major issue is the radiation sensitivity of CMOS-based charge pumps in NAND arrays, which typically fail beyond 100 krad. Developing radiation-hardened charge pumps is thus essential for fully radiation-hardened FeFET SSDs. These findings establish laminated FeFETs as a promising platform for future radiation-hardened 3D NAND technologies, combining a large memory window, retention stability, and superior radiation resilience.

## Supplementary Material



## Notes

Detailed fabrication process flow (section S1), HRTEM and EDS images of the FE oxide (section S2), and ferroelectric characterization (section S3) (PDF)
