# Supplementary material for: Enabling Radiation Hardness in Solid-State NAND Storage Utilizing a Laminated Ferroelectric Stack
Source: Nano Lett. 2026 Mar 5;26(10):3390–7. doi: 10.1021/acs.nanolett.5c05947 (PMC13003478; doi:10.1021/acs.nanolett.5c05947)
Supplement: Supplementary file 1 [file nl5c05947_si_001.pdf]

Supporting information

Enabling Radiation-Hardness in Solid-State NAND Storage Utilizing Laminated Ferroelectric Stack

AUTHOR NAMES

Lance Fernandes[1], Stuart Wodzro[1], Prasanna Venkatesan[1], Priyankka Ravikumar[1], Ming-Yen Lee[1], Minji Shon[1], Dyutimoy Chakraborty[1], Taeyoung Song[1], Sanghyun Kang[1], Sa lma Soliman[1], Mengkun Tian[2], Jason Yeager[3], Jackson Adler[3], Jiayi Chen[1], Zekai Wa ng[1], Douglas Wolfe[3], Shimeng Yu[1], Andrea Padovani[4], Suman Datta[1], Biswajit Ray[5], Asif Khan[1]

AUTHOR ADDRESS

[1]School of Electrical and Computer Engineering, Georgia Institute of Technology, Atlanta, GA, 30332, USA;

[2] Institute of Matter and Systems, Georgia Institute of Technology, Atlanta, GA, 30332, USA;

[3]Department of Material Science and Engineering, Pennsylvania State University, PA, USA;

[4]Department of Engineering Sciences and Methods, University of Modena and Reggio Emilia, Reggio Emilia, Italy;

[5]Department of Electrical and Computer Engineering, Colorado State University, CO, USA;

Table of Contents

S1: Fabrication process flow of poly-Silicon channel laminated Fe-FET

S2: Cross-Section TEM Images with EDS mapping of laminated gate stack

S3: Ferroelectric characterization of laminated metal-ferroelectric-metal (MFM) capacitor stacks before and after irradiation

S1: Fabrication process flow of poly-Silicon channel Laminated FeFET

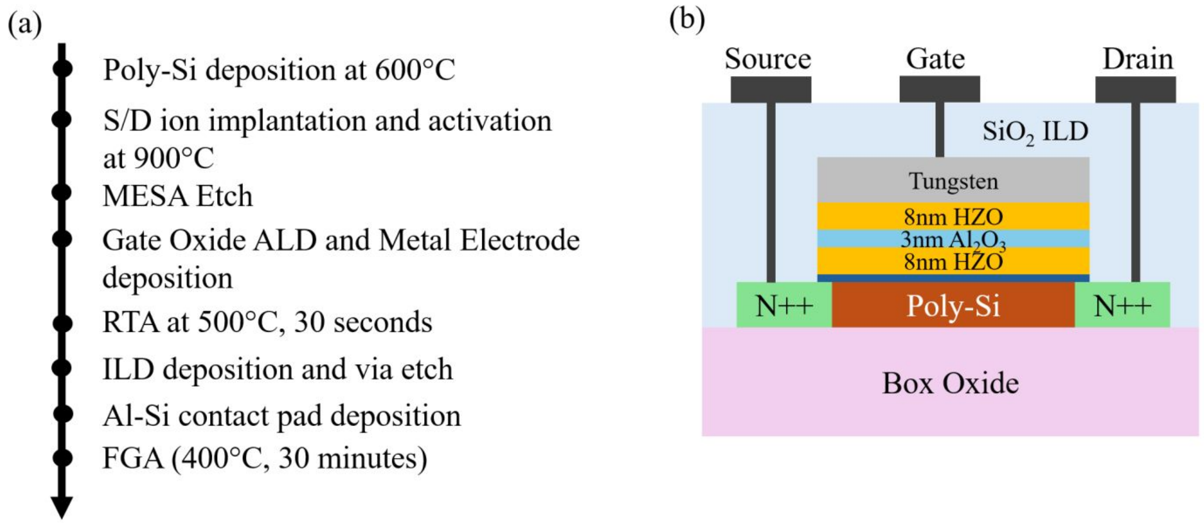

Figure S1. (a) Process flow for the laminated Fe-FET on poly-Si channel (b) Schematic cross-section of the laminated FEFET device.

The fabrication process begins with the deposition of a 40 nm poly-Silicon channel layer on a SiO_2 substrate at 600 ˚C. The source and drain regions are heavily implanted with phosphorus dopants, followed by an activation anneal at 900 ˚C for 20 minutes. The poly-Si layer is then etched to define the channel mesa. After an SC1 cleaning step, the gate oxide is deposited by thermal ALD at 250 ˚C, forming an 8 nm HZO / 3 nm Al_2O_3 / 8 nm HZO laminated stack. The Hafnium and Zirconium precursor is TDMAH (Tetrakis Dimethylamino Hafnium) and TDMAZ (Tetrakis Dimethylamino Zirconium) respectively. The Aluminum precursor is TMA (Trimethylaluminum). This is followed by deposition of a 30 nm tungsten gate electrode and subsequent gate patterning. The SC1 clean simultaneously generates a thin chemical SiO_2 layer that serves as the channel interfacial layer (dark blue layer in Fig. S2b). The gate stack is annealed at 500 ˚C for 30 seconds to induce ferroelectricity. Next, a 400 nm SiO_2 interlayer dielectric (ILD) is deposited, followed by via patterning and dry etching. Finally, Al–Si contact

pads are deposited, and a forming gas anneal at 400 ˚C for 30 minutes is performed to improve channel interface quality.

S2: Cross-Section TEM Images with EDS mapping of laminated gate stack

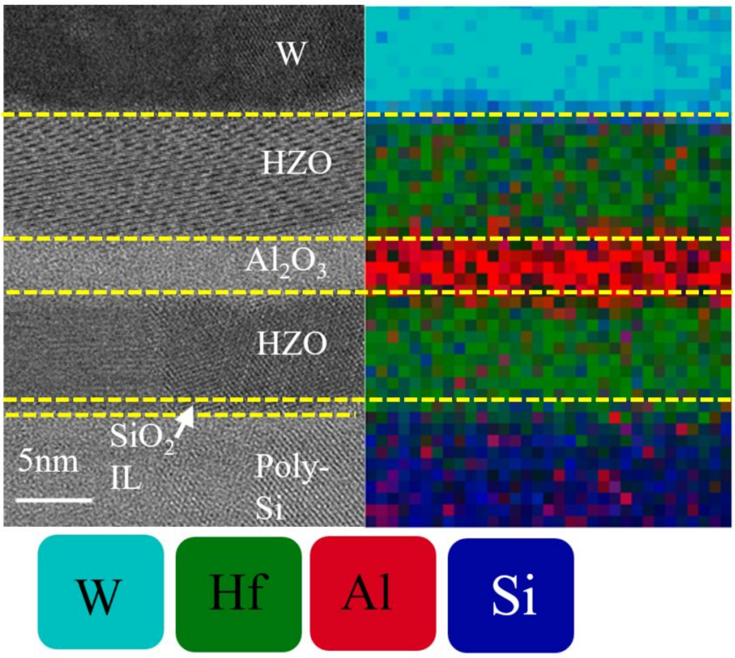

Figure S2. TEM image of the laminated FE-NAND stack on poly-Si channel with EDS elemental mapping for each layer.

Fig. S2 shows the TEM image of the laminated FE oxide stack on poly-Si channel. The HZO layers are crystallized as seen from the TEM image. The EDS elemental mapping shows each layer in the laminated stack.

S3: Ferroelectric characterization of laminated metal-ferroelectric-metal (MFM) capacitor stacks before and after irradiation

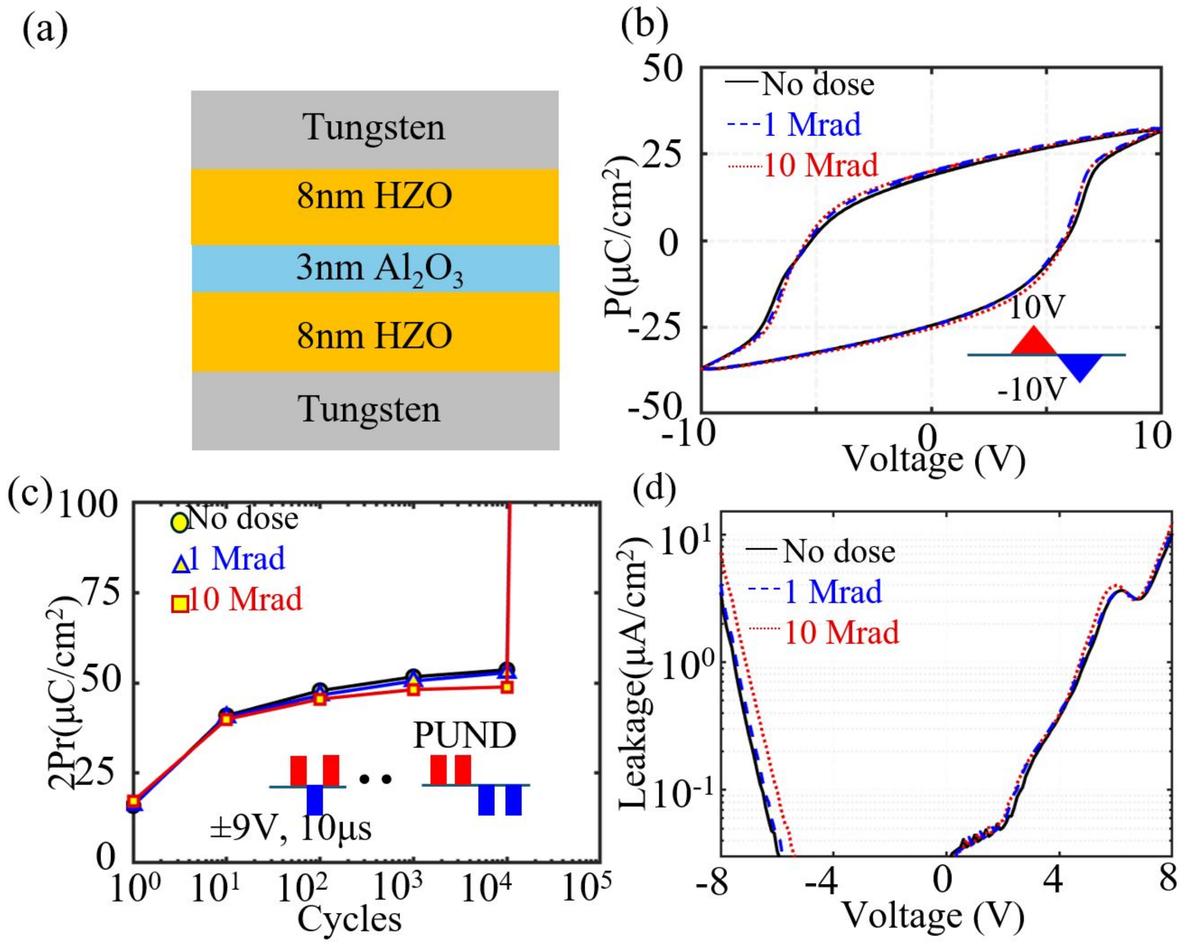

Figure S3.1. (a) Schematic of laminated metal-ferroelectric-metal (MFM) capacitor stacks used for characterizing ferroelectric properties (b) P–V hysteresis loops from MFM capacitors. (c) 2P□ values extracted from positive-up-negative-down (PUND) measurements as a function of cycling for control (unirradiated) and irradiated MFM capacitor samples. (d) Leakage current characteristics of laminated stack on MFM capacitors for control (unirradiated) and irradiated case.

To evaluate TID effects on ferroelectric properties, metal–ferroelectric–metal (MFM)

capacitors (Fig. S3.1a) were irradiated at 1 and 10%Mrad(air). Fig.%S3.1b shows P–V curves with a stable coercive voltage ($2V_c$) of 11%V and no polarization degradation even at 10 Mrad(air) TID. Fig.%S3.1c presents remnant polarization ($2P_r$) from positive-up-negative-down (PUND) measurements as a function of bipolar cycles, increasing from 16 to 50%μC/cm²

due to the wake-up effect, consistent across all doses. The effect of TID on bipolar cycling is minimal with no change in $2P_r$ or wake-up effect. Fig.%S3.1d shows DC I–V on laminated stack to estimate the leakage current. No significant change in leakage current with TID was seen. This suggest robust ferroelectric properties of laminated stack with minimal change in leakage and polarization even at extreme doses of 10 Mrad.

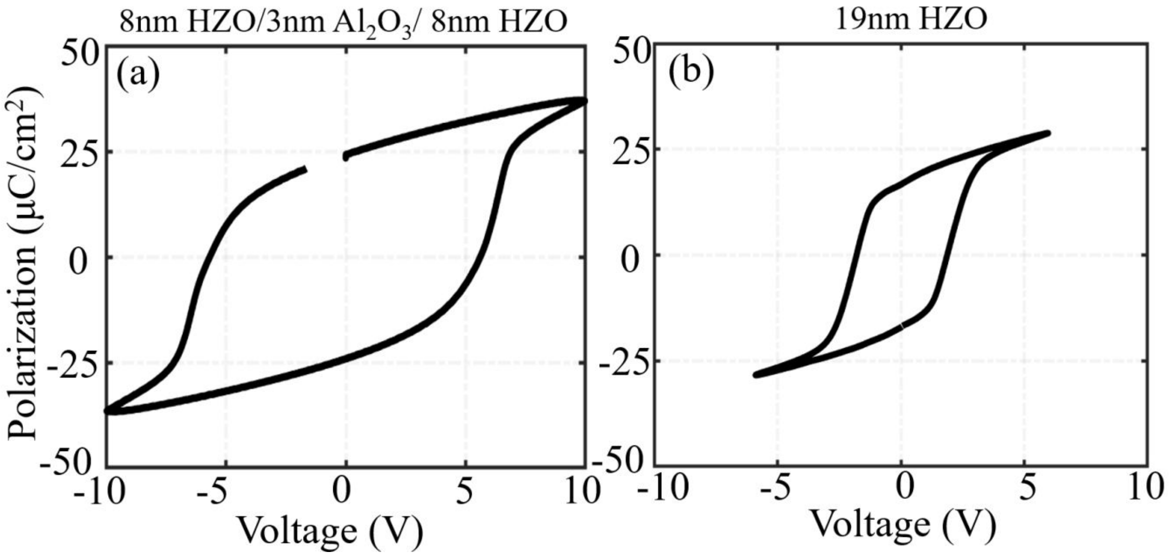

Figure S3.2. (a) P-V characteristics of Laminated stack and (b) HZO-only stack of equal physical thickness on MFM capacitor.

Fig. 3.2(a-b) shows the P-V curves extracted from MFM capacitors under full switching condition for Laminated stack and reference 19nm HZO-only stack. As seen in Fig. 3.2(a-b), the $2P_r$ is 1.5x larger in laminated stack compared to reference HZO-only stack of equal

thickness. Moreover, the coercive voltage ($2V_c$) is roughly 3x larger than HZO-only stack suggesting the ferroelectric properties are improved in laminated stack.
